# Assessment of Serum UCH-L1 and GFAP in Acute Stroke Patients

**DOI:** 10.1038/srep24588

**Published:** 2016-04-14

**Authors:** Changhong Ren, Firas Kobeissy, Ali Alawieh, Na Li, Ning Li, Kazem Zibara, Susie Zoltewicz, Joy Guingab-Cagmat, Stephen F. Larner, Yuchuan Ding, Ronald L. Hayes, Xunming Ji, Stefania Mondello

**Affiliations:** 1 Institute of Hypoxia Medicine, Xuanwu Hospital, Capital Medical University, Beijing, 100053, China; 2 Beijing Key Laboratory of Hypoxia Conditioning Translational Medicine, Beijing, 100053, China; 3 Center of Stroke, Beijing Institute for Brain Disorder, Beijing 100069, China; 4 Department of Psychiatry, Center for Neuroproteomics and Biomarkers Research, University of Florida, Gainesville, Florida, USA; 5 Department of Biochemistry and MolecularGenetics, Faculty of Medicine, American University of Beirut Medical Center, Beirut, Lebanon; 6 Department of Microbiology and Immunology, Medical University of South Carolina, Charleston, SC 29425, USA; 7 Faculty of Medicine, American University of Beirut Medical Center, Beirut, Lebanon; 8 Biology Department, Faculty of Sciences, Lebanese University, Beirut, Lebanon; 9 Banyan Labs, Banyan Biomarkers Inc., Alachua, FL, USA; 10 Department of Neurosurgery, Wayne State University, School of Medicine, Detroit, 48201, MI, USA; 11 Department of Biomedical, Dental and Morphological and Functional Imaging Sciences, University of Messina, Messina, Italy

## Abstract

A rapid and reliable diagnostic test to distinguish ischemic from hemorrhagic stroke in patients presenting with stroke-like symptoms is essential to optimize management and triage for thrombolytic therapy. The present study measured serum concentrations of ubiquitin C-terminal hydrolase (UCH-L1) and glial fibrillary astrocytic protein (GFAP) in acute stroke patients and healthy controls and investigated their relation to stroke severity and patient characteristics. We also assessed the diagnostic performance of these markers for the differentiation of intracerebral hemorrhage (ICH) from ischemic stroke (IS). Both UCH-L1 and GFAP concentrations were significantly greater in ICH patients than in controls (p < 0.0001). However, exclusively GFAP differed in ICH compared with IS (p < 0.0001). GFAP yielded an AUC of 0.86 for differentiating between ICH and IS within 4.5hrs of symptom onset with a sensitivity of 61% and a specificity of 96% using a cut-off of 0.34ng/ml. Higher GFAP levels were associated with stroke severity and history of prior stroke. Our results demonstrate that blood UCH-L1 and GFAP are increased early after stroke and distinct biomarker-specific release profiles are associated with stroke characteristics and type. We also confirmed the potential of GFAP as a tool for early rule-in of ICH, while UCH-L1 was not clinically useful.

Stroke is the third most common cause of morbidity and disability worldwide^1^. Although extensive effort in clinical and translational research has been directed at developing new therapies for stroke, the intravenous recombinant tissue-plasminogen activator (rtPA) remains the only FDA approved pharmacological therapy with a very narrow therapeutic window (3–5 hours) after onset of ischemic stroke (IS). Consequently, in patients with a suspected stroke it is critical to establish a rapid and accurate diagnosis and reliably distinguish IS from intracerebral hemorrhage (ICH) and stroke mimics, to optimize triage for thrombolytic therapy, avoid unnecessary medications or procedures and, ultimately, improve patient outcome^2^,^3^.

To date, brain imaging remains the gold standard for differentiating patients with IS and ICH and is routinely conducted in the initial assessment of stroke. However, there are some associated limitations, particularly within the first hours after a stroke. Computerized tomography (CT) accurately identifies cases of ICH, but is relatively insensitive in detecting acute and small IS. On the other hand, although magnetic resonance imaging (MRI) and in particular diffusion-weighted imaging (DWI) has emerged to undoubtedly improve stroke detection (~95%), some infarcts may not appear for several days, and some may never become visible^4^,^5^. In addition, MRI with diffusion may not be feasible in patients unstable or with contraindications, and the availability of this expensive equipment and technology is limited to specialized hospitals with experienced neuroradiologists.

Similar to other diseases including myocardial infarction in which blood biomarkers have been widely integrated into clinical management a blood biomarker-test associated with ischemic or hemorrhagic stroke may represent a valuable adjunct to current routinely available diagnostic methods and provide an objective cost-effective and rapid tool approach for early diagnosis, triage, and prognosis of stroke patients^6^, and ultimately for guiding specific pathobiology-based therapeutic interventions. An ideal diagnostic marker of stroke should exhibit the following features: 1. brain specificity; 2. diagnostic accuracy; 3. reproducibility; 4. being rapidly measurable early after injury in blood samples at a reasonable cost; and 5. offering unique powerful complementary information to guide medical decision making (clinical utility)^7^.

Identification of reliable biomarkers for stroke is under intensive investigation in preclinical and clinical studies. Among the several candidates that have been proposed glial fibrillary acidic protein (GFAP) and ubiquitin C-terminal hydrolase L1 (UCH-L1) hold significant promise.

GFAP is a brain-specific astrocytic intermediate filament protein found almost exclusively in the central nervous system (CNS)^8^. A number of clinical studies have explored the use of serum GFAP as a tool for diagnosis and prognosis in traumatic brain injury (TBI) and stroke^9^,^10^,^11^ UCH-L1 is a cytoplasmic deubiquitinating enzyme of neurons, highly enriched in CNS^12^, that has been associated with synaptic plasticity and homeostasis and to the brain’s self-repair mechanisms after injury^13^,^14^. Like GFAP, numerous experimental and clinical studies have shown increased UCH-L1 levels in cerebrospinal fluid (CSF) and blood of patients following TBI and stroke. In addition, significant correlations with disease severity and outcome have been observed^15^,^16^,^17^.

Importantly, because of their own distinct features and cellular origins UCH-L1 and GFAP allow to assess and explore cell-type–specific injury patterns and different pathophysiological mechanisms in brain injury^18^,^19^. In addition, their dual combination is under an extensive analytical and clinical validation^16^,^20^,^21^,^22^ and holds the most promise for point-of-care (POC) application that can entails a very rapid transferability to the clinical practice.

Given the potential of these 2 markers to unveil important aspects of stroke pathophysiology, the paucity of available data in the literature and the likely availability of information to physicians at the bedside in a very near future, we decided in the present study to investigate the simultaneous assessment of GFAP and UCH-L1 in patients presenting with stroke-like symptoms and their relation to initial stroke severity and patient characteristics. We also evaluated the diagnostic performance of these biomarkers alone and in combination for the differentiation between IS and ICH.

## Results

### Population

The study population included a total of 45 ICH, 79 IS, 5 subarachnoid hemorrhage (SAH) and 3 transient ischemic attack (TIA) patients and 57 controls. Baseline demographic and clinical characteristics of patients and controls are listed in Table 1, showing that they were well matched with regard to all key characteristics.

### Serum Concentrations of UCH-L1 and GFAP

**Figure 1:**
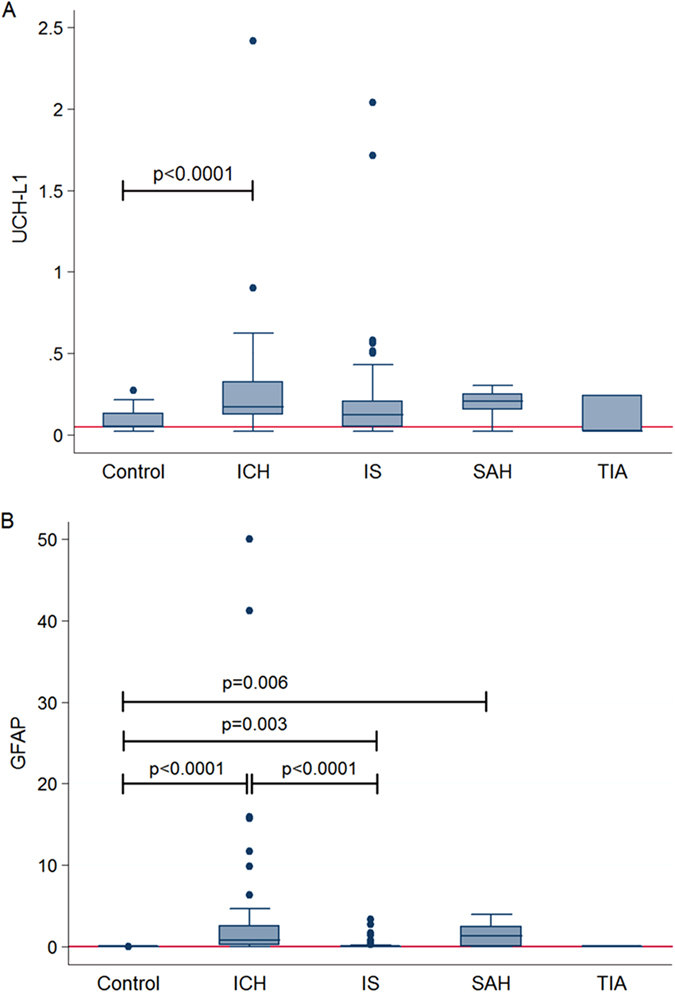
Box-and-whisker plots demonstrating UCH-L1 and GFAP concentrations within 24 hours of symptom onset. Serum UCH-L1 **(A)** and GFAP concentrations **(B)** in patients with stroke or TIA and controls. The horizontal line in each box represents the median, with the boxes representing the interquartile range. Significant differences are indicated (Kruskal-Wallis test). The reference line (red line) represents the median of estimated normal values.

**Figure 2:**
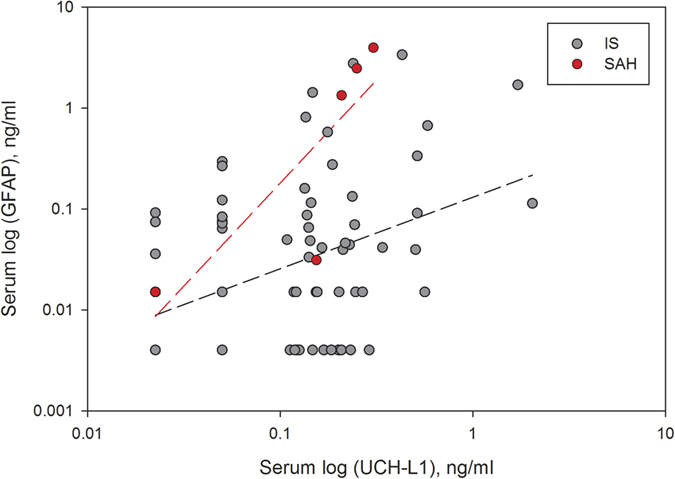
Correlations of logged serum GFAP and UCH-L1 concentrations in patients with IS and SAH (r = 0.36 and r = 1.00, respectively, test based on the Spearman’s rank correlation).

The median serum concentrations of UCH-L1 and GFAP for patients (stroke or TIA) and controls are shown in Table 2. Serum UCH-L1 was significantly higher in patients with ICH compared to controls (p < 0.001) (Fig. 1) and tended to be higher in SAH and IS patients. Serum GFAP concentrations were significantly higher in patients with ICH, IS and SAH compared to controls, but concentrations were similar when comparing TIA patients with controls. GFAP concentrations were also found to be significantly elevated in patients with ICH compared to IS patients (Table 2 and Fig. 1). UCH-L1 concentrations were weakly correlated with GFAP in IS patients (r = 0.36, p = 0.001), and strongly correlated in SAH patients (r = 1.00, p = 0.017) (Fig. 2). No similar correlations were found in ICH or TIA cases. No correlation between time to sample withdrawal and biomarker level was found.

### Correlation of UCH-L1/GFAP levels and NIHSS in stroke patients

In IS patients, individual GFAP values correlated with the corresponding NIHSS score (r = 0.30, p = 0.007), but no other correlations were found. Based on the NIHSS score on admission, patients were classified as mild (NIHSS ≤ 7) or moderate to severe stroke patients (NIHSS > 7), as previously described^23^. In ICH patients there were no differences in UCH-L1 and GFAP levels between mild and moderate-severe stroke patients (data not shown). IS patients with mild stroke had significantly lower levels of GFAP than patients with moderate to severe stroke (0.015 vs 0.07 ng/ml, p = 0.009), while UCH-L1 did not differ between the two subpopulations.

### UCH-L1/GFAP levels in relation to history of stroke

The median serum UCH-L1 in ICH patients was higher in patients with no history of stroke compared to those who had a previous stroke (0.26 vs 0.13 ng/ml, respectively, p = 0.008); however, GFAP concentrations did not differ between these two subpopulations. On the other hand, in IS patients, the median serum GFAP level was significantly lower in subjects with no history of stroke compared to those with a previous stroke (0.015 vs 0.07 ng/ml, respectively, p = 0.004), while there was no difference in the levels of UCH-L1 between these 2 subgroups.

### ROC curve analyses

**Figure 3:**
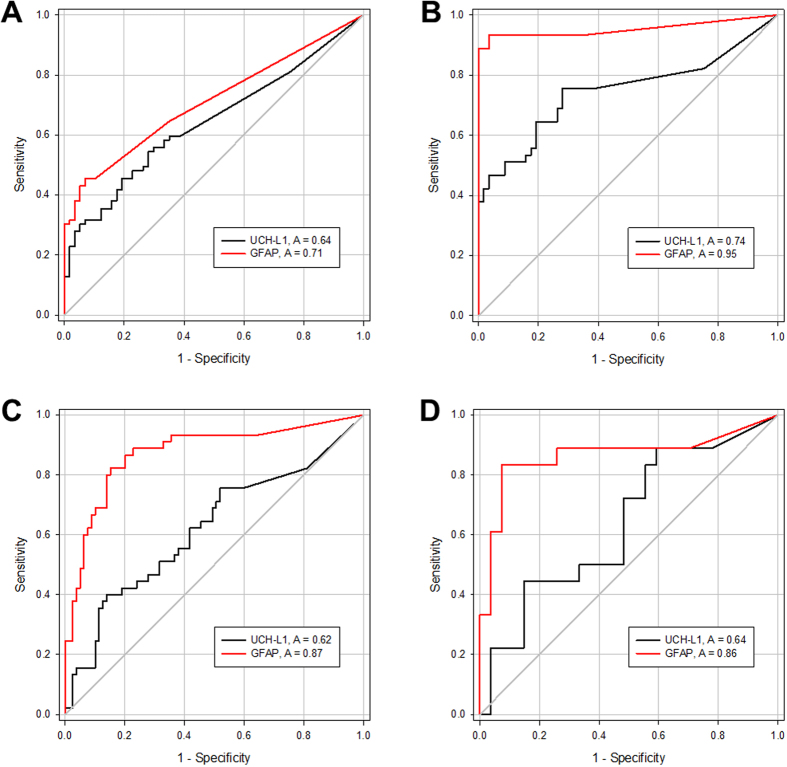
ROC curves for UCH-L1 (black line) and GFAP (red line) in serum for distinguishing patients with IS (A) and ICH (B) from controls, and for differentiating between patients with IS and patients with ICH at various time points (C) and within 4.5 hours of symptom onset (D). The area under the curves is indicated.

ROC curve analyses was performed and demonstrated that UCH-L1 and GFAP levels were able to distinguish patients with IS from controls, with an area under the curve of 0.64 (95% CI 0.55 to 0.73) and 0.71 (95% CI 0.63 to 0.79) (Fig. 3A), respectively. Furthermore, UCH-L1 and GFAP levels were able to distinguish patients with ICH from controls, with an area under the curve of 0.74 (95% CI 0.64 to 0.85) and 0.95 (95% CI 0.90 to 1.00) (Fig. 3B), respectively.

The area under the curve of UCH-L1 and GFAP for discriminating between IS and ICH patients were 0.62 (95% CI 0.51 to 0.72) and 0.87 (95% CI 0.80 to 0.94), respectively. There was a significant difference in diagnostic accuracy between these markers (p < 0.001, test based on the Mann-Whitney statistic) (Fig. 3C). More specifically, using a cut-off point of 0.34 ng/ml GFAP was able to differentiate patients with IS from those with ICH with a sensitivity of 0.67 and a specificity of 0.91. The combination of UCH-L1 and GFAP did not improve diagnostic accuracy (AUC 0.875) compared with GFAP alone.

Additionally, as we were specifically interested in using neuronal and glial markers in serum as a tool to rule out ICH in patients with ischemic stroke who are eligible for acute thrombolytic therapy, we evaluated the diagnostic accuracy of UCH-L1 and GFAP measurement within 4.5 hours of symptom onset. Diagnostic accuracy was essentially similar to that calculated using the entire dataset (UCH-L1, AUC 0.64 [95% CI 0.47 to 0.81]; GFAP, AUC 0.86 [95% CI 0.73 to 0.99]) (Fig. 3D). Using a cut-off point of 0.34 ng/ml GFAP was able to differentiate patients with IS from those with ICH with a slightly improved specificity (sensitivity 0.61, specificity 0.96).

### Multiple logistic regression analyses for GFAP concentrations

Univariate binary logistic regression analysis showed that several characteristics were strongly associated with higher GFAP concentrations following acute stroke (Table 3). Multivariate logistic regression analysis of patients with stroke, including all significant variables, only identified ‘previous history of stroke’ and ‘NIHSS’ as independent predictors of higher GFAP concentrations (Table 4). Consistently, multivariate logistic regression analysis revealed that previous history of stroke and NIHSS were found to independently contribute to the probability of having higher GFAP concentrations when only patients with ischemic stroke were considered (Table 4). Conversely, these variables did not appear to independently influence GFAP concentrations in patients with ICH.

## Discussion

The major finding of this study is that acute ICH patients had significantly higher serum levels of both GFAP and UCH-L1 as compared to matched healthy controls, and that serum GFAP but not UCH-L1 was significantly lower in patients with IS compared with those with ICH. Importantly, we also showed for the first time that higher GFAP levels were associated with history of previous episode of stroke. Furthermore, our data provide additional evidence that circulating GFAP has high discriminatory power for the clinically relevant differential diagnosis of ICH versus IS, while UCH-L1 does not appear to be clinically useful in this context.

In this controlled prospective study, we explored two candidate biomarkers for early diagnosis of stroke, namely UCH-L1 and GFAP, which are abundantly expressed in neuronal and glial cell respectively. These two protein biomarkers have not been simultaneously assessed in prior studies on human stroke subjects evaluating and comparing their diagnostic performance. In particular, we found that UCH-L1 was substantially higher in ICH cases than in healthy volunteers, distinguishing patients with ICH from controls, with an accuracy of 0.74; while there was no significant difference between IS patients and healthy controls. This finding probably reflects a more sudden and instantaneous necrotic neuronal death and disruption of the BBB that occurs following ICH. However, Liu *et al.* reported that serum UCH-L1 level was also significantly elevated in experimental ischemic stroke model^7^. In line with this investigation, our previous study has demonstrated that UCH-L1 was elevated after an ischemic but not hemorrhagic stroke in rats^15^. The discrepancy between human and animal studies is unclear, but it may reflect differences between rodents and humans in the release kinetics of brain injury biomarkers, regulation of blood circulation and clearance or ability to repair the BBB following ischemia^7^.

A number of clinical research studies explored the use of serum GFAP as a tool for the diagnosis and prognosis prediction in TBI and stroke patients^24^. Our results confirmed that levels of GFAP from patients with acute ICH were significantly elevated compared to those with IS and that GFAP can reliably distinguish ICH from IS with a high specificity indicating the potential utility of this test to accurately rule-in ICH particularly very early after symptom onset. This ability of GFAP to rule-in ICH can be especially valuable in the pre-hospital setting when specific interventions such as lowering of increased blood pressure or the rapid reversal of anticoagulation can be applied and be most effective^25^. Our findings are in accordance with previously published studies^9^,^10^,^26^,^27^ showing that GFAP is an effective biomarker for acute stroke differential diagnosis and reporting similar diagnostic accuracy. Nonetheless, there is a large variation in the thresholds used for diagnosis (from 0.11 to 4 ng/ml) across studies, which may generate concern about a clinical use. Many reasons may explain this variability including characteristics of the patients, differences in sample type (plasma vs. serum) and time points as well as lack of standardization, analytical factors (batch-to-batch variations of the ELISA kits, laboratory equipment and procedures) and absence of validated reference methods. Future rigorous and standardized quality control studies are required for identification and harmonization of optimum cutoff values.

Another aim for this work was to evaluate the use of GFAP in combination with UCH-L1 for the differential diagnosis of ICH and IS. Surprisingly, the diagnostic accuracy of GFAP was not increased when combined with UCH-L1. However, Unden *et al.* reported that the sensitivity of GFAP is increased once combined with activated protein C-protein inhibitor complex^27^. Several studies have shown that inflammatory mediators and acute-phase response markers (C-reactive protein [CRP], interleukin 6 [IL-6], tumor necrosis factor alpha [TNF-α], matrix metalloproteinase 9 [MMP-9])^28^, components of the coagulation system and hemostasis (thrombomodulin, D-dimer, fibrinogen, and von Willebrand factor [vWF])^29^,^30^,^31^,^32^, markers of lipid peroxidation (malondialdehyde [MDA])^33^ and adipocyte fatty acid-binding protein (A-FABP)^34^ appear to identify the ischemic nature of stroke. Therefore, a multimarker approach based on the combination of GFAP and these biomarkers that are differently altered across ischemic and hemorrhagic stroke might prove to be of clinical value providing complementary and incremental information and might better distinguish the disease entities and increase sensitivity and specificity compared with individual markers. Future studies are warranted to identify and validate a blood-based biomarker panel for the diagnosis, characterization and stratification of stroke patient population for routine use in clinical practice.

Noteworthy, the levels of both UCH-L1 and GFAP were not found elevated in the TIA group. This finding might be explained by the fact that TIA triggers a cascade of molecular mechanisms and neurometabolic events that not necessarily result in evident structural changes and parenchymal damage to the brain and therefore may not be detectable using glial or neuronal proteins in serum. Other markers of microvascular damage, thrombosis and neurotoxicity may more appropriately capture pathophysiological mechanisms underlying TIA and are currently under extensive investigation as surrogate markers for TIA diagnosis^35^,^36^.

In addition to the difference in biomarker levels across the patients groups, we showed that UCH-L1 concentrations correlated with GFAP level in patients with IS and SAH, but no other correlations were found. This may be attributable to the different cell origin and protein characteristics (e.g. molecular weights [UCH-L1 25 kDa and GFAP ~50 kDa]) as well as the distinctive pathophysiology and tissue damage associated with different types of strokes which may affect the temporal dynamics and passage across the blood brain barrier (BBB) thereby resulting in distinct biomarker-specific release patterns. Previous experimental and human studies of biomarker kinetics from our group support this hypothesis^37^,^38^. More recently, we also demonstrated variability in biomarker profiles (UCH-L1 and GFAP) across different experimental TBI models and provided clear evidence that biomarker levels and time course are associated to the overall magnitude of injury and BBB disruption severity as well as different types of injuries and locations^39^. Further studies are urgently needed to determine potential stroke-signature patterns of brain damage biomarkers.

In this study, we also investigated the correlation of UCH-L1 and GFAP levels with patient characteristics including age, gender, diabetes, hyperlipidemia and having a previous history of stroke. An intriguing observation arising from these analyses is that ‘history of previous stroke’ was independently associated with higher GFAP concentrations after acute ischemic stroke. This finding fits in well with a seminal study from Dietrich’s group demonstrating that a first initial cerebrovascular event induces a state of brain vulnerability which predisposes the brain to more severe extensive damage after a second ischemic insult^40^. This increased vulnerability appears to be likely a consequence of persistent pathophysiological processes including vascular disturbances, long-term alteration of BBB permeability and damage to the brain parenchyma itself triggered by the initial stroke. For clinical practice, it would be extremely helpful if GFAP may serve as a marker for risk stratification in patient with stroke by characterizing and quantifying the ongoing chronic damage and possibly helping to determine a temporal window of brain vulnerability occurring after the initial insult. Future studies are warranted.

Although no significant differences in the serum levels of GFAP and UCH-L1 in SAH samples compared with IC and ICH were observed, GFAP levels after SAH were higher compared to controls, which is consistent with previous clinical studies^41^. Nonetheless, because of the limited available data, more investigation is needed before drawing any conclusions on whether GFAP may be used in the diagnosis and as a guide for medical decisions in SAH patients.

In this study, we focused on analysis of two biomarkers reflecting different types of structural damage and pathophysiological mechanisms thereby providing complementary information. However, due to the complex pathophysiology of stroke and the vast spectrum of molecular events triggered by the initial insult, UCH-L1 and GFAP cannot be sufficient and it will be necessary to broaden our arsenal to cover other significant pathophysiological mechanisms that come into play including inflammation, oxidative stress, axonal injury and remodeling and molecular reorganisation of membrane and extracellular-matrix proteins. This is likely to the most successful strategy that will lay the foundation of personalized therapeutic approach in stroke. However, the two biomarkers investigated in this study can be of critical value in subsequent studies that combine these biomarkers to other potential candidates with the ultimate aim of providing a reliable, rapid and accurate panel of blood biomarkers to stratify and predict outcome of stroke patients.

Some limitations of this study should be acknowledged. The main limitation is the relatively modest sample size which may affect the reproducibility of our results^42^. Future large multi-center studies are necessary to confirm our findings and determine the true clinical utility of these biomarkers in the management of patient with a suspected stroke. Further, we did not assess differences in the level of UCH-L1 and GFAP based on stroke location. It is likely that biomarker concentrations will differ based on the ischemic location in the human brain (e.g. cortical vs. subcortical infarct), as suggested by previous studies^23^. This will be an important avenue for future investigation. Another limitation of the study was the lack of outcome information, which would have helped to elucidate the relationships of initial biomarker levels and recovery patterns. However, in the present study, we were especially interested in characterizing the diagnostic value of UCH-L1 and GFAP and their potential in the acute care setting.

In summary, we demonstrated elevation of blood UCH-L1 and GFAP early after acute stroke and distinct biomarker-specific release patterns associated with stroke type and characteristics contributing to the current knowledge on the pathophysiology and the role of brain damage markers in acute stroke. Finally, our data also provide important additional evidence that serum GFAP shows promising diagnostic value for detecting ICH in acute stroke patients.

## Methods

### Subjects

This study was reviewed and approved by the Xuanwu Hospital, Capital Medical University Institutional Review Board, and a written informed consent was obtained from patients or legal representatives. The study was carried out in accordance with the approved guidelines.

One hundred seventy-seven patients admitted with acute stroke or transient ischemic attack to the Emergency Department of Xuanwu Hospital, Capital Medical University and Department of Neurology of Beijing Renhe Hospital, China, were enrolled in this study. Inclusion criteria were sudden occurrence of a focal neurological deficit secondary to ICH, SAH, IS or TIA, admission within 24 hours of symptom onset, presence of neurological symptoms at the time of admission, and adequate access to patient information. Standard definitions of TIA and stroke in accordance with guidelines were used^43^. All patients underwent standard neurological and general medical evaluation and assessment using the National Institutes of Health Stroke Scale (NIHSS). Past medical history and medication history were obtained. History of prior strokes was based upon medical record documentation, and findings on imaging studies. ICH, SAH or IS were diagnosed using initial or consecutive brain imaging (CT or MRI).

Controls consisting of healthy volunteers who did not have any focal neurological deficit and antecedents of central nervous system disease were enrolled from the Medical Examination Center of Xuanwu Hospital, Capital Medical University, China.

### Blood sample collection and processing

At hospital admission, 2mL of blood was collected from each subject by venipuncture into gel-separator tubes for serum (BD Company). Blood tubes were rapidly transported to the laboratory facility of the hospitals and centrifuged at 1500 *g* for 10 minutes within 10 to 60 minutes after blood collection. Serum was then separated in 0.5 ml aliquots and stored at −80°C. The samples were shipped on dry ice to Banyan Biomarkers Inc.

### Enzyme-linked immunosorbent assay

Serum samples were processed by board-certified laboratory technicians who were blinded to clinical information. Proprietary sandwich enzyme-linked immunosorbent assays (ELISAs) were used to determine the concentrations and temporal profiles of UCH-L1 and GFAP in the serum samples. The employed GFAP assay is able to detect full-length as well as GFAP break down products (BDPs). Banyan has successfully used these sensitive biomarker assays in a series of previously published studies in adults with TBI and neurodegenerative diseases^21^,^44^. Briefly, both mouse monoclonal rabbit polyclonal antibody against recombinant UCH-L1 full length and partial protein, were produced in-house at Banyan Biomarkers, Inc. Similarly, a proprietary mouse monoclonal antibody for solid phase immobilization and a polyclonal rabbit detection antibody were used for ELISA, to detect the levels of intact GFAP and its BDPs. This approach allows a more sensitive detection of GFAP in patients’ blood^21^,^22^. Standard curves using recombinant proteins were generated for each assay and quantitative determination of the biomarker levels in the de-identified samples were based on four-parameter non-linear regression analyses using SigmaPlot version 11 (Systat, Chicago, IL, USA).

### Statistical Analyses

Data normality was assessed using the Kolmogorov–Smirnov test. Results are presented as mean (±SD) or median (interquartile range) as appropriate. The Mann–Whitney U-test was used to assess differences in biomarker concentration between 2 groups and the Kruskal-Wallis test was used to assess the overall differences in biomarker concentration across three or more groups. For age, the only normally distributed continuous variable, the one-way analysis of variance (ANOVA) was used. Correlation analyses between biomarker serum concentrations and quantitative variables were performed using the nonparametric Spearman rank correlation test. The association between categorical variables was investigated using the chi-square or Fisher’s exact test, as appropriate.

Receiver operating characteristic (ROC) curve analysis was used to calculate diagnostic accuracy of biomarkers for distinguishing between stroke and controls and between ICH and IS and to determine the optimal cutoff with optimized sensitivity and specificity for the identification of ICH. The overall measure of diagnostic accuracy of the models was assessed using the area under the receiver operating characteristic curve (AUC). The AUC is a measure of predictive discrimination with the value of one representing perfect accuracy and 0.5 representing a random guess.

Univariate logistic regression was applied to determine whether demographic and clinical characteristics of stroke patients (age, sex, diabetes mellitus, hyperlipidemia, previous history of stroke, NIHSS Score) independently influenced GFAP concentrations. GFAP as the dependent variable was categorized according to the optimal identified cutoff. Variables associated with outcome in the univariate analysis (p < 0.05) were included in additional multivariable logistic regression models to determine factors that could be considered independent risk factors for increased GFAP concentrations. Adjusted odds ratios are reported with their respective 95% CIs. We evaluated several models due to collinearity of candidate variables.

Data were analyzed according to the pre-specified statistical analysis plan. Two-sided tests were used and a p-value < 0.05 was considered significant. Statistical analysis was performed using the SAS software version 9.2 (SAS Institute Inc., Cary, NC, USA).

## Additional Information

**How to cite this article**: Ren, C. *et al.* Assessment of Serum UCH-L1 and GFAP in Acute Stroke Patients. *Sci. Rep.* **6**, 24588; doi: 10.1038/srep24588 (2016).

## Figures and Tables

**Table 1:** Baseline characteristics of healthy volunteers and patients with stroke or TIA.

	Healthy Volunteers (n = 57)	ICH (n = 45)	IS (n = 79)	SAH (n = 5)	TIA (n = 3)	P value
Age, years, mean (SD)	58.93 (9.82)	58.91 (12.18)	61.1 (13.33)	63.08 (10.55)	46.67 (6.51)	0.171
Gender, n (%)						
Female	27 (47.37)	9 (45)	30 (37.98)	2 (40)	1 (33.33)	0.46
Male	30 (52.63)	36 (80)	49 (62.02)	3 (60)	2 (66.67)	
NIHSS on admission, median (interquartile range)	NA	7 (4–11)	4 (1–8)	0 (0–2)	0	*0.008
Time to sampling from symptom onset, h, median (interquartile range)	NA	7 (3–24)	10 (4–24)	12 (3–24)	24 (3–24)	0.81
Patients with hyperlipidemia, n (%)	19 (33.33)	10 (22.22)	24 (30.38)	1 (20)	1 (33.33)	0.76
Patients with diabetes, n (%)	9 (15.79)	5 (11.11)	17 (21.52)	1 (20)	1 (33.33)	0.41
History of Previous Stroke, n (%)	7 (12.28)	13 (28.89)	23 (29.11)	0	0	0.07

NA, not applicable.

**Table 2:** Median serum concentrations of UCH-L1 and GFAP in patients (stroke or TIA) at the time of hospital admission and in controls.

	Healthy Volunteers (n = 57)	ICH (n = 45)	IS (n = 79)	SAH (n = 5)	TIA (n = 3)	P value^a^
UCH-L1	0.05 (0.02–0.13)	0.17 (0.09–0.35)	0.13 (0.05–0.21)	0.21 (0.09–0.28)	0.02 (0.02–0.24)	<0.0001
GFAP	0.004 (0.004–0.02)	0.81 (0.18–3.31)	0.02 (0.004–0.08)	1.33 (0.02–3.21)	0.004 (0.004–0.09)	<0.0001

Data are given as median (interquartile range).

^a^Kruskall-Wallis test.

**Table 3:** Crude OR with 95% confidence intervals of clinical and demographic variables for higher GFAP concentrations (dichotomized according to the identified optimal cut-off value), using univariate logistic regression.

	GFAP concentrations (categorized according to the optimal cut-off (≤0.036 ng/ml vs >0.036 ng/ml)		
Variable	OR (95%CI)	p	C
Age	1.027 (0.998–1.057)	0.07	0.61
Gender		0.14	0.57
Female	Reference		
Male	1.802 (0.832–3.901)		
NIHSS Score	1.206 (1.087–1.337)	0.004	0.70
Diabetes			
No	Reference		
Yes	2.724 (1.060–6.997)	0.037	0.58
Hyperlipidemia			
No	Reference		
Yes	1.340 (0.601–2.988)	0.48	0.53
Previous history of stroke			
No	Reference		
Yes	4.67 (1.77–12.34)	0.002	0.64

C = The area under an ROC curve (also known as c-statistic) provides an overall measure of diagnostic accuracy, with the value of one representing perfect accuracy.

OR = odds ratios; GFAP = glial fibrillary acidic protein; NIHSS = NIH Stroke Scale/Score.

**Table 4:** Crude OR with 95% confidence intervals of clinical and demographic variables for higher GFAP concentrations (dichotomized according to the identified optimal cut-off value), using univariate logistic regression.

	GFAP concentrations (categorized according to the optimal cut-off (≤0.036 ng/ml vs >0.036 ng/ml)	
Variable	OR (95%CI)	C
All patients with stroke (ICH and IS)		
NIHSS Score	1.204 (1.082–1.339)†	0.77
Previous history of stroke		
No	Reference	
Yes	4.845 (1.701–13. 805)^‡^	
Patients with IS		
NIHSS Score	1.143 (1.015–1.287)*	0.79
Previous history of stroke		
No	Reference	
Yes	8.261 (2.494–27.367)†	

C = The area under an ROC curve (also known as c-statistic) provides an overall measure of diagnostic accuracy, with the value of one representing perfect accuracy.

*p < 0.05, ^‡^p < 0.01, ^†^p < 0.001.

OR = odds ratios; GFAP = glial fibrillary acidic protein; NIHSS = NIH Stroke Scale/Score.
